# Performance Spectrum of Home-Compostable Biopolymer Fibers Compared to a Petrochemical Alternative

**DOI:** 10.3390/polym15061372

**Published:** 2023-03-09

**Authors:** Simon Schick, Robert Groten, Gunnar H. Seide

**Affiliations:** 1 Aachen-Maastricht Institute for Biobased Materials (AMIBM), Faculty of Science and Engineering, Maastricht University, Brightlands Chemelot Campus, Urmonderbaan 22, 6167 RD Geleen, The Netherlands; 2 Department of Textile and Clothing Technology, Niederrhein University of Applied Sciences, Campus Mönchengladbach, Webschulstrasse 31, 41065 Mönchengladbach, Germany

**Keywords:** biopolymers, biodegradable fiber, melt-spinning, polypropylene, thermoplastic starch, polybutylene succinate, polybutylene adipate terephthalate, crystallinity

## Abstract

Manufacturers of technical polymers must increasingly consider the degradability of their products due to the growing public interest in topics such as greenhouse gas emissions and microplastic pollution. Biobased polymers are part of the solution, but they are still more expensive and less well characterized than conventional petrochemical polymers. Therefore, few biobased polymers with technical applications have reached the market. Polylactic acid (PLA) is the most widely-used industrial thermoplastic biopolymer and is mainly found in the areas of packaging and single-use products. It is classed as biodegradable but only breaks down efficiently above the glass transition temperature of ~60 °C, so it persists in the environment. Some commercially available biobased polymers can break down under normal environmental conditions, including polybutylene succinate (PBS), polybutylene adipate terephthalate (PBAT) and thermoplastic starch (TPS), but they are used far less than PLA. This article compares polypropylene, a petrochemical polymer and benchmark for technical applications, with the commercially available biobased polymers PBS, PBAT and TPS, all of which are home-compostable. The comparison considers processing (using the same spinning equipment to generate comparable data) and utilization. Draw ratios ranged from 29 to 83, with take-up speeds from 450 to 1000 m/min. PP achieved benchmark tenacities over 50 cN/tex with these settings, while PBS and PBAT achieved over 10cN/tex. By comparing the performance of biopolymers to petrochemical polymers in the same melt-spinning setting, it is easier to decide which polymer to use in a particular application. This study shows the possibility that home-compostable biopolymers are suitable for products with lower mechanical properties. Only spinning the materials on the same machine with the same settings produces comparable data. This research, therefore, fills the niche and provides comparable data. To our knowledge, this report is the first direct comparison of polypropylene and biobased polymers in the same spinning process with the same parameter settings.

## 1. Introduction

Petrochemical polymers such as polypropylene (PP), polyethylene terephthalate (PET) and polyamide (PA) are widely used to manufacture melt-spun fibers and have been produced for many years. These fibers are used in clothing and disposable items such as wet wipes and filters. Given the widespread use of such polymers, a large body of research data has accumulated. As early as 1986, the correlation between shear in the production process and the orientation of polymer fibers was understood for a wide range of petrochemical polymers [1]. Spinnability is a key aspect of material selection that contributes to the subsequent performance of the fiber, reflecting the density, tenacity, glass transition temperature and melting temperature of the polymer. Other parameters, such as flame retardancy, resistance to chemicals, UV light and abrasion, have been studied in depth because they are relevant for textile applications. These parameters have been investigated individually or in combination for all the common petrochemical polymers, and some examples are presented in Table 1. PP, PA and PET have good tenacity, chemical resistance and abrasion resistance, but UV resistance is “sufficient” for PA and PET and poor for PP [2]. PP, PA and PET fibers achieve tenacities of up to 82 cN/tex [3,4].

In addition to physical properties, the degradability of polymers is an important consideration due to the growing public interest in microplastic pollution and greenhouse gas emissions. Single-use articles are normally classed as organic waste and are either incinerated or consigned to landfills, but accidental or improper disposal causes such items to accumulate in the environment. However, home composting is increasingly viewed as a favorable end-of-life scenario for single-use products. Polymers are home-compostable when 90% of the material can decompose at 28 °C within 12 months [12]. The home composting of single-use articles satisfies the principle of proximity and has other benefits such as reducing the need to process mixed materials in recycling [13].

One of the key strategies to reduce the environmental accumulation of plastics is a switch from petroleum-based polymers to biopolymers that break down naturally. Polylactic acid (PLA) is one of the most widely used biopolymers because it can be composted under industrial conditions (>60 °C) with careful moisture management [2], but it has a low biodegradability under normal environmental conditions and is not suitable for home composting [12]. PLA currently represents 18.9% of the European biopolymers market [14], but alternatives such as polybutylene adipate terephthalate (PBAT, 19.2%), thermoplastic starch (TPS, 16.4%) and polybutylene succinate (PBS, 3.5%) are compatible with home composting. The biopolymer market from 2021 to 2026 is predicted to shift significantly, with PBAT rising to 30% and PBS rising to 16%, while PLA falls to 10.4% and TPS to 5.2% [14]. The most promising home-compostable biopolymers for single-use products therefore appear to be PBAT, PBS and TPS, although starch cannot be spun alone and is therefore blended with PBAT [12].

Despite the promising markets for these alternative and more compostable biobased polymers, there is much less information available to support their applications compared to PLA. TPS is processed as a compound because starch alone does not have good thermoplastic behavior [15]. Several blends have been investigated, such as TPS with polyvinyl butyrate, revealing that starch processing is hindered by the loss of molar mass at higher shear forces in the extruder, although excellent tensile strength was achieved in blends containing 22% starch [16]. When PBAT is spun in a BiCo process with PBT, the spinnability improves compared to the single polymer. With a 10,000 m/min take-up speed, the thermal and mechanical behavior of the fibers was enhanced [17]. PBAT was also spun into fibers with a take-up speed of 5000 m/min, which improved the molecular orientation, crystal structure, and mechanical properties [18]. A blend of PBAT and PLA was spun with graphene, the latter influencing the degradability and enhancing the mechanical properties [19]. PBS was spun as monofilaments and drawn at different speeds and temperatures to produce fishing gear [20]. It was also blended with PLA to increase the crystallinity, making the PLA fibers more ductile and increasing the washing and rubbing fastness for healthcare applications [21]. PBS and microfibrillated cellulose (MFC) significantly improved the tensile strength of fibers at a high draw ratio [22]. In a BiCo process, PLA/PBS and PLA/PP fibers were compared, revealing that the PLA/PP fibers could be split but the PLA/PBS fibers could not [23].

Our literature analysis has shown that home-compostable biobased polymers are only rarely spun alone but more often in a blend or as a BiCo process. For example, monofilaments were spun out but drawn in a second step, or low take-up speeds were utilized, which are not common industrial practices. Lack of knowledge about home-compostable biopolymers’ performance, especially in comparison with petrochemical alternatives, discourages companies from considering them as alternatives to petrochemical polymers. This results in lower usage, especially for single-use products like packaging or hygiene articles. We are not aware of any comparison between home-compostable biopolymers and petrochemical polymers using the same industrial spinning process. Therefore, it is imperative to investigate further the potential for home-compostable biopolymers to compete with petrochemical polymers in production processes. We, therefore, characterized three different home-compostable biopolymers (PBS, PBAT and TPS) processed on an industrial melt-spinning machine and compared them to a PP standard that was tested and spun using the same methods. The biopolymers and petrochemical alternatives were compared to the fiber requirements determined at the beginning of the experiment.

## 2. Materials and Methods

### 2.1. Materials

PBS (PBS_FZ_71PB), PBAT (R4923 Ecoflex F Blend C1200 PBAT) and TPS (R2708 AGRANA ARIC 4007, a TPS/PBAT blend where the quantities of the components are not specified) were supplied by the International Fibers Group ASOTA (Linz, Austria). 

### 2.2. Thermogravimetric Analysis (TGA)

The mass loss over the temperature range 0–700 °C was determined using a Q500 device (TA Instruments, Asse, Belgium) with a heating rate of 10 °C/min and a nitrogen flow of 50 mL/min. The temperatures of 5% and 50% mass loss were determined using TA Universal Analysis 2000.

### 2.3. Differential Scanning Calorimetry (DSC)

A Q2000 device (TA Instruments) was used to determine the melting temperature (T_m_) and crystallinity of the polymers. The T_m_ was evaluated by monitoring the behavior of each polymer over the temperature range −30 to 220 °C (PBS, PBAT and PP) or −30 to 200 °C (TPS) at a fixed heating rate of 10 °C/min. The crystallinity was determined after spinning by monitoring the behavior of each polymer in three heating cycles of −30 to 200 or 220 °C as above. The data were analyzed using TA Instruments Universal Analysis 2000. Melt enthalpy values for 100% crystalline polymers ($\Delta H_{m}^{100}$) were taken from the literature. The crystallinity achieved during the spinning process was then calculated using Equation (1), where ΔH_m_ is the melt enthalpy. 

Equation (1), calculation of crystallinity:

(1) $$X_{C}=\frac{\Delta H_{m}}{\Delta H_{m}^{100}}*100$$

### 2.4. Rheology

The rheological properties of the polymers were determined by using a DHR1 rheometer (TA Instruments) to conduct a frequency sweep, amplitude sweep and temperature sweep. For the amplitude sweep, the temperature was set to T_m_ + 20 °C and the angular frequency to 10 rad/s for all polymers. The amplitude, or strain, varied from 1% to 10%. In the frequency sweep, the temperature was set to T_m_ + 20 °C and the strain to 1% for all polymers. The angular frequency varied from 1 to 628 rad/s. In the temperature sweep, the frequency was set to 10 rad/s and the strain to 1%. The set temperatures of T_m_ + 20 °C, T_m_ + 30 °C, T_m_ + 40 °C, T_m_ + 50 °C and T_m_ + 60 °C were then selected for the measurements.

### 2.5. Gel Permeation Chromatography (GPC)

The relative molecular mass and molecular mass distribution of each polymer (except PP) were measured by gel permeation chromatography (GPC) using a 1260 Infinity System (Agilent Technologies, Santa Clara, CA, USA). The polymers were dissolved in a mobile phase consisting of hexafluoro-2-isopropanol (HFIP) with 0.19% sodium trifluoroacetate. The flow rate during the test was 0.33 mL/min. GPC analysis was carried out before and after the rheology tests. We focused on the influence of temperature and shear stress on the molecular mass of the biopolymers. 

### 2.6. Melt Spinning 

**Figure 1:**
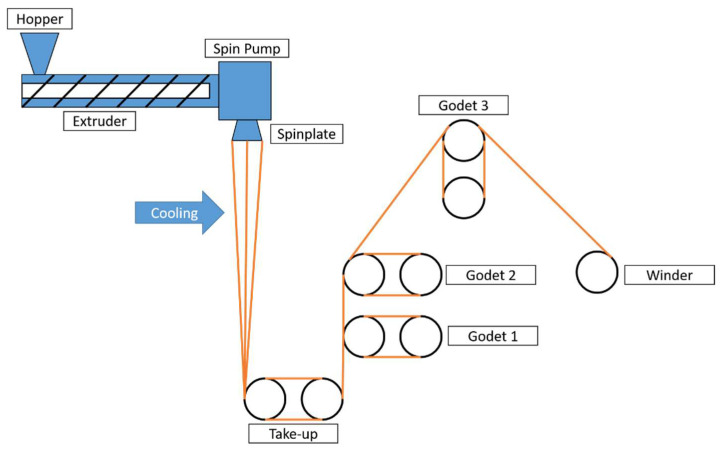
The spinning line of the FET-100 device.

The polymer granules were dried in a vacuum at 80 °C overnight before spinning on a FET-100 device (Fiber Extrusion Technology, Leeds, UK) with a single-screw extruder at 60 bar extruder pressure (Figure 1. The spinning line).

The extruder temperature profiles, throughput, cooling air settings, take-up speeds, roller pair temperatures (godets), and drawing ratios are summarized in Table 2. The fibers were spun using a 48-hole spin plate with a 0.25-mm nozzle diameter and 0.5-mm nozzle length (48H 0.25 × 0.5). Some of the PP samples were spun using a 48H 0.3 × 0.6 spin-plate. The overall draw ratio (ODR) is shown as a combination of the melt draw ratio (MDR) and the draw ratio (DR). The MDR is the ratio of the exit speed at the nozzle to the take-up speed. The DR describes the relationship between the take-up godet and the winder. We selected take-up speeds of 400–500 m/min, 750 m/min and 1000 m/min. Higher take-up speeds or drawing ratios were only used if lower settings resulted in a stable process. Due to the set take-up speeds, the speed of the first pair of godets was set and the remaining three were fine-tuned to ensure a stable spinning process. Winder speeds were set to achieve draw ratios of 1.1, 1.5, 2.0, 2.5, 3.0, etc. As a result, the winder speeds were adjusted to achieve the desired result, but minor deviations could occur due to fine-tuning. The fiber tension between the individual godets and between godet four and the winder was monitored using a digital tension meter. The fibers were wound onto bobbins at 10 cN. The industrial winder WinTens 602 (STC Spinnzwirn, Chemnitz, Germany) was used at speeds of 500–4200 m/min for 5–10 min.

The comparison of the different polymers can be achieved by choosing the same parameters. Therefore, a low take-up speed was selected to sustain a stable spinning process. The spinning speed was increased only when a stable process was realized for several minutes.

### 2.7. Tensile Test

Tensile force and elongation were determined using a ZwickLine Z2.5 device (Zwick Roell, Ulm, Germany) with an Xforce HP cell for 50 N and capstan clamps for fiber testing. With a clamping length of 100 mm and a pre-tension of 0.1 cN/tex, the tests were carried out at 200 mm/min based on DIN EN ISO 5079. The linear density (titer) was determined gravimetrically based on DIN EN ISO 1973. The measured titer was divided by 48 (48 nozzles in the spinneret) to obtain the single fiber titer.

## 3. Results and Discussion

Based on three individual measurements, each measurement was averaged. In addition to the measurement inaccuracy, which is inevitable with any measuring equipment, each measurement is statistically proven. With more measurements, this averaged value would further demonstrate the accuracy.

### 3.1. TGA Measurements

**Figure 2:**
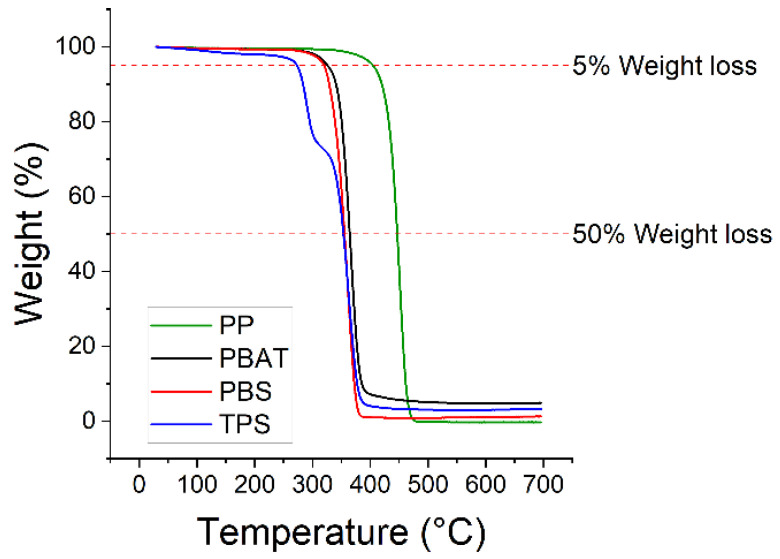
Thermograms showing the temperatures of 5% and 50% mass loss for four polymers.

The temperatures of 5% and 50% mass loss were determined by TGA (Table 3). The corresponding thermograms are shown in Figure 2. 

The highest temperatures of 5% and 50% mass loss were observed for PP. PBS and PBAT showed similar values, which were much lower than PP. However, the curves of all three polymers were similar in shape. In contrast, TPS presented a unique two-step curve that converged on those of PBS and PBAT after ~40% mass loss. This may reflect the evaporation of water present within the starch component of the blend, which was already mentioned in reference [24]. One-step degradation has been reported for several other petrochemical polymers, including polycaprolactone [25]. 

### 3.2. GPC Analysis

**Figure 3:**
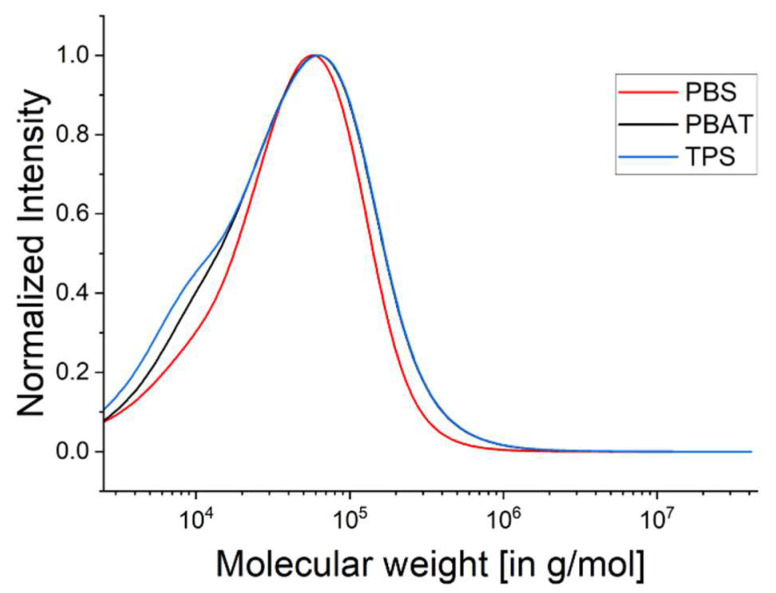
GPC elugrams before rheological measurement representing the biopolymers PBS, PBAT and TPS.

The molecular weight, molecular weight distribution and polydispersity of the biopolymers (but not PP) were determined by GPC analysis (Table 4). The corresponding elugrams are shown in Figure 3. The curves representing all three biopolymers include some initial noise (between 100 and 200 g/mol), which may reflect the molecular weight of HFIP (168.05 g/mol) and/or the test equipment. The TPS curve has a shoulder on the left side of the peak, which can also be seen in the lower M_n_ value compared to the other polymers. The PBAT curve has a steep incline on the left side of the peak, which can also be seen in the higher M_n_ value compared to the other polymers. 

**Figure 4:**
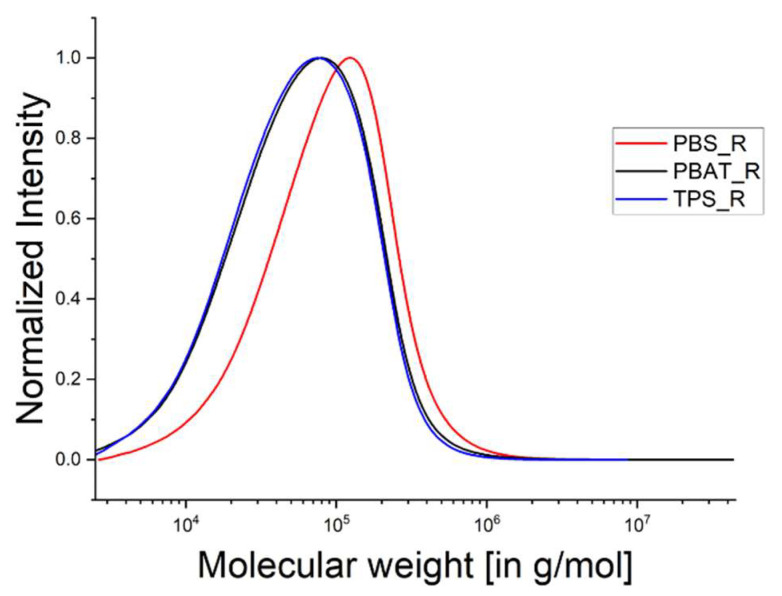
GPC elugrams after rheological measurement representing the biopolymers PBS, PBAT and TPS.

The traces of all three biopolymers were very similar before rheological measurement (Figure 3). The same measurements were taken after rheology (Table 5), and the corresponding elugrams are shown in Figure 4. The polymers were subject to shear stress and high temperature in the rheometer similar to the conditions in the extruder during the spinning trial, which lasted 15–20 min.

We observed only slight differences in the GPC traces before and after rheology, with a somewhat smoother appearance in the second set of readings and, in the case of PBS, a slight increase in M_n_. The TPS and PBAT curves overlap and the PBS curve is narrower than the others. TPS also lost the shoulder on the left side of the peak. All three curves also shifted to the right. PBS showed the most significant shift towards a higher M_w_, which may be caused by chain extension due to the dwell time in the process [26]. A general increase in M_w_ and M_n_ was observed, along with a decline in polydispersity, which may reflect the chain extension of all three polymers. 

### 3.3. Rheology Measurements

**Figure 5:**
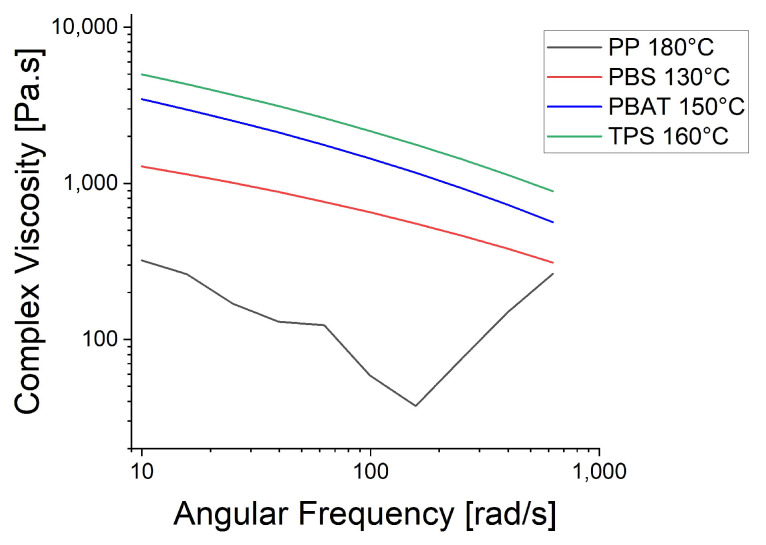
Rheology of four polymers at T_m_ + 20 °C.

Rheology measurements at T_m_ + 20 °C revealed that TPS showed the highest complex viscosity among the biopolymers and PBS the lowest (Figure 5). All four polymers showed shear thinning behavior as the angular frequency increased. This is linked to polymer chain entanglement, reflecting the Van der Waals forces between the chains, which increase the viscosity at low shear rates. If the shear rate is increased, the Van der Waals forces break up, and the viscosity decreases because the chains move more freely. The nature of the PP curve can be explained by the slippage of polymer chains between the plates. 

**Figure 6:**
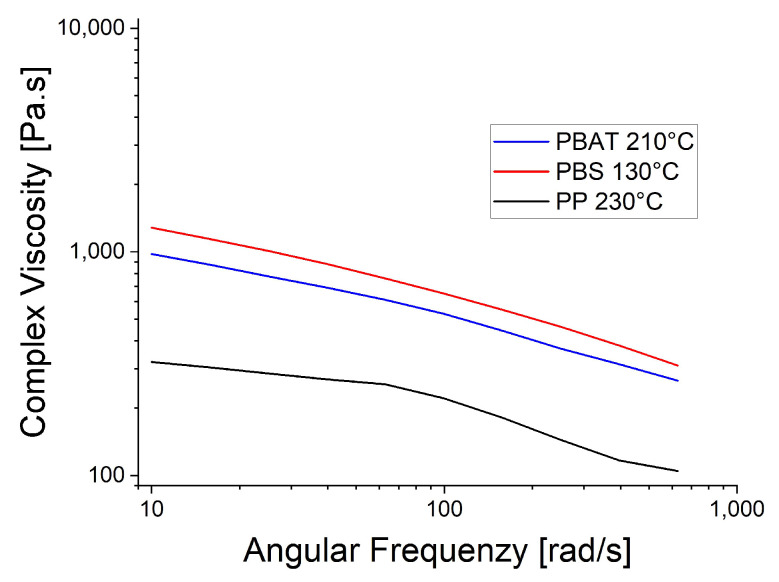
Rheology of three biopolymers at the spinning temperature.

**Figure 7:**
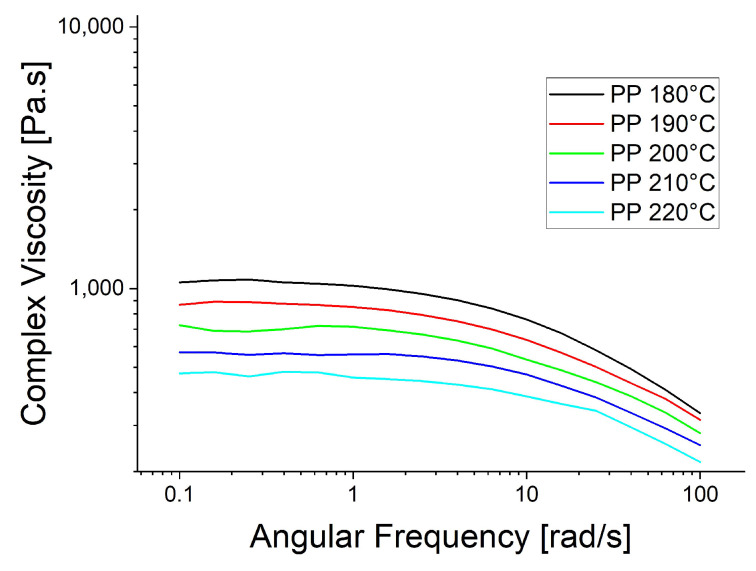
Rheological temperature sweep for PP.

**Figure 8:**
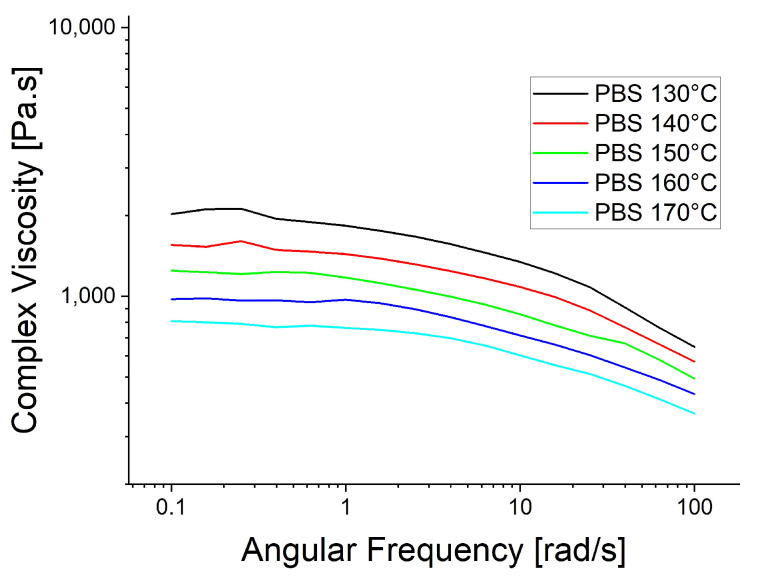
Rheological temperature sweep for PBS.

**Figure 9:**
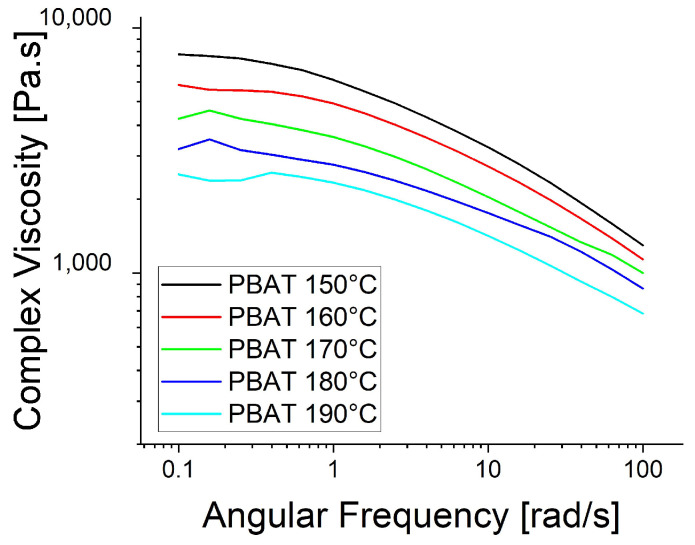
Rheological temperature sweep for PBAT.

**Figure 10:**
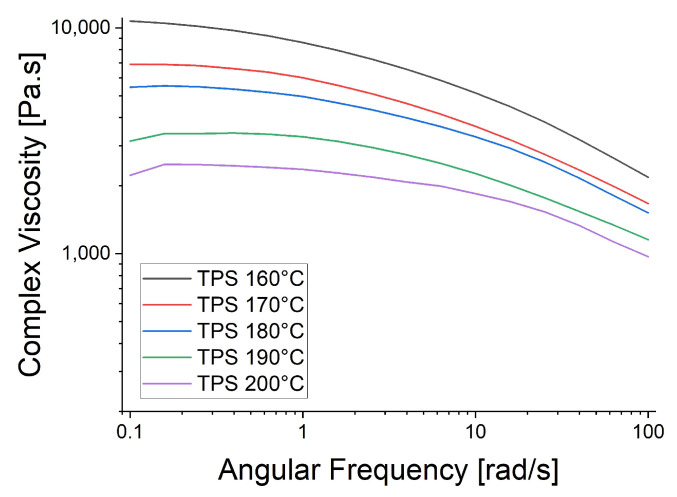
Rheological temperature sweep for TPS.

The rheological trials were repeated at the spinning temperatures: 130 °C for PBS, 210 °C for PBAT and 230 °C for PP (Figure 6). The higher temperatures reduced the viscosity of PBAT and PP, causing the PP curve to become smoother, supporting the proposed polymer slippage at T_m_ + 20 °C for PP. At the lower temperatures (Figure 5), the complex viscosity of PBAT declined more rapidly than that of PBS as the angular frequency increased. In contrast, the two polymers showed similar declines in complex viscosity when the temperature of PBAT was increased (Figure 6). We also carried out a temperature sweep at T_m_ + 20 °C, T_m_ + 30 °C, T_m_ + 40 °C, T_m_ + 50 °C and T_m_ + 60 °C. The complex viscosity is shown as a function of angular frequency at the different temperatures for PP in Figure 7, PBS in Figure 8, PBAT in Figure 9 and TPS in Figure 10.

The temperature had the greatest impact on TPS, resulting in the largest difference in viscosity between 160 and 200 °C (Figure 10). As above, shear thinning was observed for all four polymers. The PP curve at 180 °C was smoothest, possibly due to the lower angular frequency used in the measurement, which might be too low for slippage (Figure 7). The melt reaches a Newtonian behavior plateau at lower frequencies but shifts towards non-Newtonian behavior at higher frequencies. 

**Figure 11:**
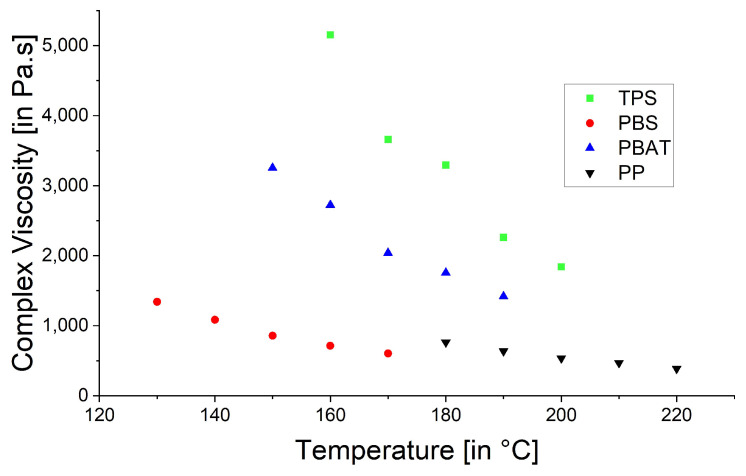
Complex viscosity as a function of temperature at 10 rad/s for four polymers.

To combine both measurements in one graph, the frequency was set to 10 rad/s and the complex viscosity was plotted against the temperature (Figure 11). The increasing temperature may break up the Van der Waals bonds, facilitating the relative movement of polymer chains. The temperature had the greatest impact on the complex viscosity of TPS, closely followed by PBAT. The temperature effect was slightly greater for PBS than PP but far below the effect observed for the other two polymers. If the increasing temperature is considered, the complex viscosity decreased at a 10 °C difference between the settings for all measurements. For the T_m_ + 20 °C to T_m_ + 30 °C interval, it was 28.97% for TPS, 16.34% for PBAT, 19.21% for PBS and 16.41% for PP. The greatest difference for the three biopolymers was observed from T_m_ + 30 °C to T_m_ + 40 °C (31.35% for TPS, 20.92% for PBS and 25.09% for PBAT), whereas the greatest difference for PP was from T_m_ + 50 °C to T_m_ + 60 °C (17.732%). The influence of temperature on viscosity has been reported previously, where stronger intermolecular bonding (in this case, higher viscosity) makes a material more prone to temperature differences [27]. This suggests that the biopolymers are more sensitive to temperature changes than PP. As the temperature increases, the intermolecular bonds between the molecules become weaker, causing the viscosity to decrease.

### 3.4. DSC Analysis and Crystallinity 

**Figure 12:**
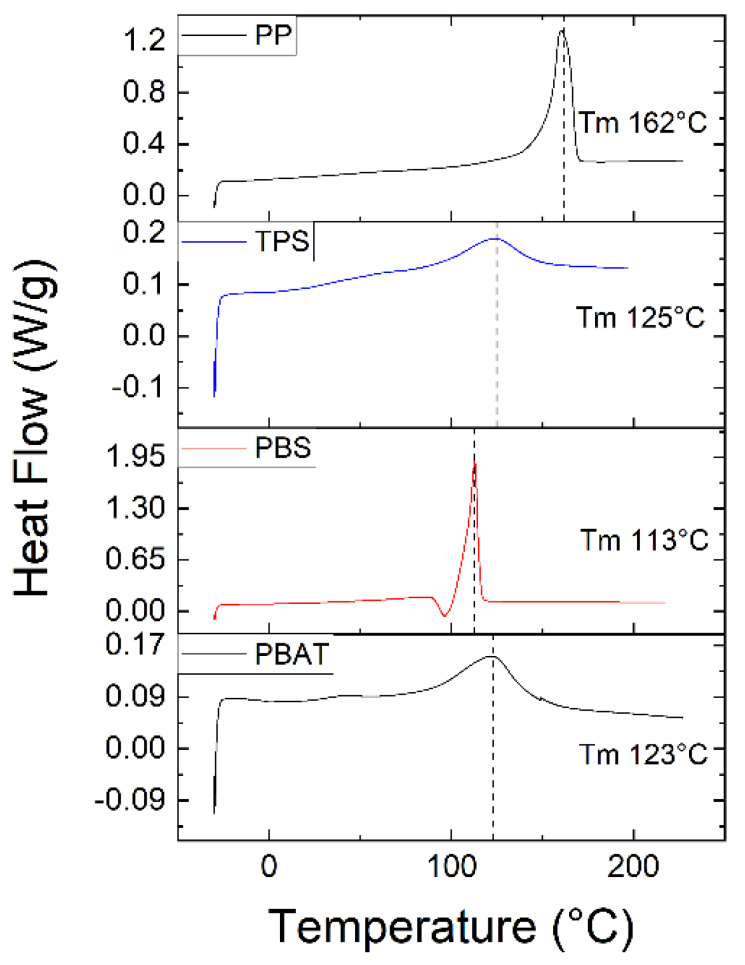
DSC analysis to determine the melting temperature (T_m_) of the four polymers.

The T_m_ of the polymer granules was determined by DSC using a heat–cool–heat cycle. The values were 162 °C for PP, 125 °C for TPS, 113 °C for PBS and 123 °C for PBAT (Figure 12). 

**Figure 13:**
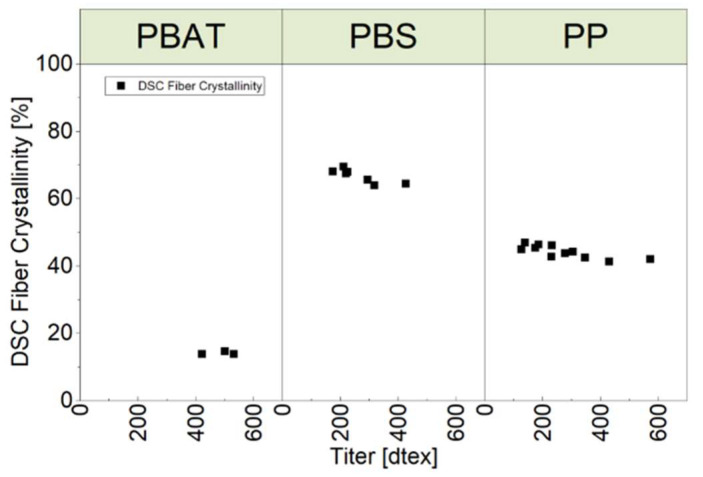
Crystallinity as a function of fiber fineness (titer in dtex) of three polymer fibers.

The melt enthalpy (ΔH_m_) was determined using a heat–cool cycle. We then combined the ΔH_m_ values with melt enthalpy values for 100% crystalline polymers (ΔH_m_^100^) in Equation (1) to determine the fiber crystallinity, which is shown as a function of fiber fineness (titer in dtex) in Figure 13. PBS achieved the highest value for crystallinity, followed by PP and PBAT. Higher crystallinities correlated with smaller titers.

### 3.5. Physical Fiber Testing

**Figure 14:**
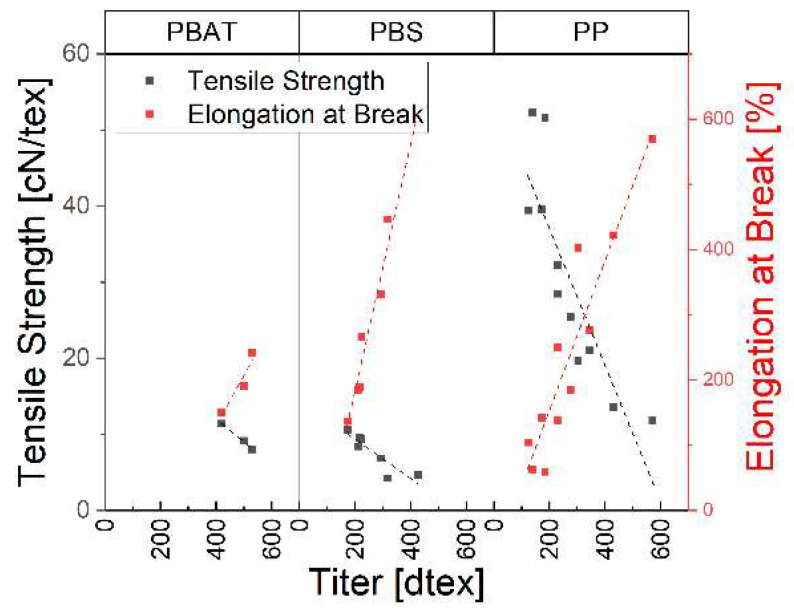
Tensile strength and elongation at break over titer for three polymer fibers.

The tenacity and elongation over titer of the three polymers are shown in Figure 14. We observed a relationship between elongation and tensile strength, with smaller fiber titers showing greater tensile strength and less elongation. Higher tensile strength in thinner fibers was accompanied by reduced elongation, indicating the importance of titer to the overall mechanical performance of the fiber. This is true for all spun materials.

### 3.6. Physical Properties and Crystallinity

**Figure 15:**
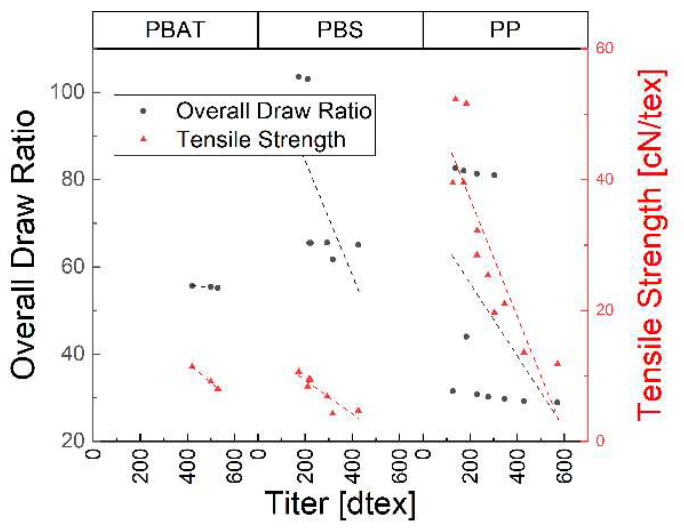
Overall draw ratio and tensile strength over fiber fineness for three polymers.

The ODR and tensile strength of the spun fibers showed similar behavior when plotted against the titer (Figure 15). This is because finer fibers are obtained at higher draw ratios and have a higher tensile strength. 

**Figure 16:**
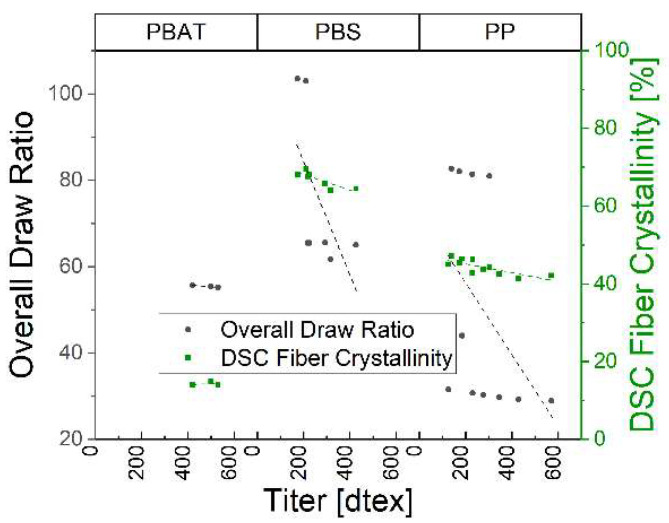
Overall draw ratio and crystallinity over fiber fineness for three polymers.

At higher ODRs, the crystallinity of all three polymers increased as the titer decreased (Figure 16). Furthermore, we expected and confirmed that the tensile strength increased with higher values of M_w_ and the tenacity increased with higher values of crystallinity, which was directly linked to a higher drawing ratio in the fiber spinning process. The ratio between disorganized and oriented polymers is between two and five [27]. Crystallinity and Mw of PBS are higher than PBAT, but the tenacity is similar. This indicates that the higher Mw of PBS leads to a higher degree of interchain entanglements, which could decrease the possibility of chain alignment in the melt-spinning process [28]. This means that the achieved tenacity of the PBS fibers could be close to the achievable maximum of PBS, whereas PBAT could be improved in future trials. The higher crystallinity of PBS also increases the alignment of the polymer chains, which is necessary for increasing the tenacity. This suggests that the spinning process might be improved in future trials for PBS and PBAT. PP showed an approximately five-fold increase in tenacity over the different draw ratios, which is commensurate with the literature values. The titer, tenacity, elongation at break, overall drawing and crystallinity of the fibers are summarized in Table 6.

Based on the literature, crystallinity should be directly linked to higher tenacities, higher draw ratios, and smaller diameters. In this spinning trial, the smallest titer with 2.87 dtex was achieved by PP, but with 47.13% crystallinity, PP is behind all crystallinity measurements for the PBS fibers. PBS, in this case, has the smallest titer of 3.6 dtex but a crystallinity of 68%. In addition, the PP11 sample shows a tenacity of 52.26cN/tex, while PBS shows 10.62cN/tex. Thus, a spun polymer with a high draw ratio has a smaller titer and a higher crystallinity. The needed draw ratio to achieve a high crystallinity for PBS differs from the draw ratio for PP for the same crystallinity, and therefore this correlation cannot be made globally.

## 4. Decision Tree Based on the Spinning Trials

**Figure 17:**
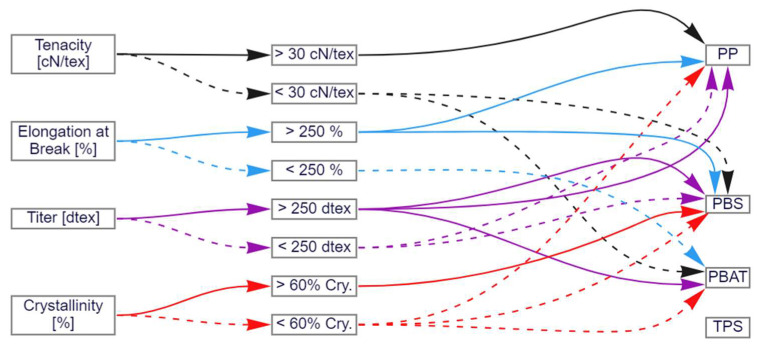
Decision tree based on the spinning trials discussed in this study.

The outcome of the spinning trials was converted into a decision tree that can be used to select polymers that are suitable to generate fibers with appropriate properties for downstream applications (Figure 17). The four main parameters are listed on the left: tenacity (black arrows), elongation at break (blue arrows), titer (purple arrows) and crystallinity (yellow arrows). The solid arrows represent high parameter values and the dashed arrows represent low values. The threshold between high (solid black) and low (dashed black) tenacity is defined at 30 cN/tex. The threshold between high (solid blue) and low (dashed blue) elongation at break is defined at 250%. The threshold between high (solid purple) and low (dashed purple) titer is defined at 250 dtex (for a 48-fiber cable). Finally, the threshold between high (solid yellow) and low (dashed yellow) crystallinity is defined at 60%. The arrows converge on the four different polymers used in this study (PP, PBS, PBAT and TPS), showing which outcomes are possible.

For example, the only polymer compatible with high-tenacity fibers is PP (solid black arrow), but lower tenacities (>10 cN/tex) are also compatible with PBS and PBAT. The decision tree can also be used in reverse to determine which properties can be expected for different polymers. 

By viewing this decision tree, it is easy to determine which material is suitable for which parameter range. Even though this study only considered a few parameters that can be tested on the spun fiber, this graphic already gives a good overview of the capabilities of the different spun polymers. The performance of the individual polymers becomes clear if this decision tree is extended with several tested parameters or polymers. Certain materials are significantly more expensive, so many companies refrain from using or considering them. If the significantly more sustainable properties outweigh the costs in a particular application area, selecting a previously unused polymer makes perfect sense. The hurdle of using a biopolymer is lowered with this study because the performance compared to a PP is made obvious.

## 5. Conclusions

While biopolymers have previously been spun on a small scale or in a two-step spinning-drawing process, but previous studies have only considered single biopolymers and have never compared biopolymers to petrochemical polymers in the same process. This is the first study to our knowledge that has compared three home-compostable biopolymers to a petrochemical polymer in an industrial-scale spinning process. This study is relevant to the industrial-scale production of biopolymers and provides valuable insight into the performance spectrum of home-compostable biopolymers compared to petrochemical polymers in the same process. The results from this study are summarized in Table 7, with the shaded cells indicating parameters derived from the melt-spinning process. We found that PBS, PBAT and TPS were able to form fibers, but only PBS and PBAT were compatible with a stable production process. 

We also characterized the spun fibers in terms of crystallinity. The machine settings achieved different fiber diameters, tenacities, elongations and crystallinities. The correlation of fine fibers with high tenacity and low elongation at break was established in all spun polymers. PBS showed a higher crystallinity than PP but was lower in tenacity and achieved the highest elongation at break. Overall, all three spinnable polymers achieved a diameter of 10 dtex or lower for the spun fibers. PBS and PBAT could be spun into fibers and achieved good tenacity, albeit lower than PP. Crystallinity is directly linked to higher tenacities, higher draw ratios and smaller diameters. The tenacities of the biopolymers were lower than PP, but their elongation at break was higher. Given that the elongation at break of the home-compostable biopolymers was higher than PP, with further process optimization their tenacities could also be improved to match PP. As mentioned, it is difficult for biopolymers to find their way onto the market. Part of the hurdle is taken by showing the performance spectrum of the various home-compostable biopolymers compared to a benchmark petrochemical polymer. When it becomes clear that fibers can be produced on the same machines with the same settings, which achieve mechanical properties for other interesting applications and degrade under home composting conditions, the material is much more accessible. As a result, petrochemical polymers are not needed in every application. Likewise, degradable fibers are only desirable in some applications. For example, fibers in hoists or safety ropes are not intended to degrade over time. In the same way, melt-binding fibers or hygiene items should only be used once and easily disposed of. 

Overall, spun home-compostable biopolymers can be used in the field of melt-binding fibers, hygiene articles, and other products that require less robust physical properties. The spun PP fibers achieved benchmark properties and can be used in narrow textiles such as lifting straps and tension belts. Our study confirmed the potential of home-compostable biopolymers compared to PP as a benchmark in an industrial-scale spinning process. 

## Figures and Tables

**Table 1:** Parameters for the physical properties of petrochemical polymers (++ very good, + sufficient, - insufficient [5,6,7,8,9,10,11].

Parameter	PP	PA	PET
Density [g/cm^3^]	0.91	1.14	1.39
Glass transition temperature [°C]	−15	50	75
Melting temperature [°C]	170	225	260
Decomposition temperature [°C]	399	387	402
Tenacity [cN/tex]	++	++	++
Chemical resistance	++	+	+
Abrasion resistance	+	++	+
UV stability	-	+	+
Flame retardancy	-	+	+

**Table 2:** FET-100 machine settings.

Sample	Spinneret	Heating Zone 1 [°C]	Spin Head Temp. [°C]	Throughput [g/min]	Cooling Air [L/min]	Take-Up [m/min]	Winding [m/min]	Fineness Single Fiber [dtex]	ODR
PBS 1	48H 0.25–0.5	120	130	17.5	300	450	500	6.4	61.71
PBS 2	48H 0.25–0.5	120	130	25	300	470	510	8.9	45.42
PBS 3	48H 0.25–0.5	120	130	17.5	300	470	750	6.3	65.92
PBS 4	48H 0.25–0.5	120	130	17.5	300	470	1000	4.7	65.48
PBS 5	48H 0.25–0.5	120	130	17.5	300	470	1000	4.5	65.48
PBS 6	48H 0.25–0.5	120	130	25	350	750	1500	4.4	72.7
PBS 7	48H 0.25–0.5	120	130	25	350	750	1850	4.6	73.2
PBAT 1	48H 0.25–0.5	185	210	25	360	400	680	10.4	39.43
PBAT 2	48H 0.25–0.5	185	210	25	360	400	575	11	39.2
PBAT 3	48H 0.25–0.5	185	210	25	360	400	755	8.8	39.7
PP 1	48H 0.25–0.5	210	230	42.5	800	500	600	11.9	28.93
PP 2	48H 0.25–0.5	210	230	42.5	800	500	750	8.9	29.24
PP 3	48H 0.25–0.5	210	230	42.5	800	500	1000	7.2	29.75
PP 4	48H 0.25–0.5	210	230	42.5	800	500	1250	5.8	30.26
PP 5	48H 0.25–0.5	210	230	42.5	800	500	1600	4.8	30.75
PP 6	48H 0.25–0.5	210	230	42.5	800	500	1750	2.6	31.52
PP 7	48H 0.3–0.6	210	230	42.5	800	500	2000	3.8	31.82
PP 8	48H 0.3–0.6	210	230	42.5	800	1000	1110	6.3	80.97
PP 9	48H 0.3–0.6	210	230	42.5	800	1000	1500	4.8	81.39
PP 10	48H 0.3–0.6	210	230	42.5	800	1000	2000	3.6	82
PP 11	48H 0.3–0.6	210	230	42.5	800	1000	2515	2.9	82.66
TPS	No stable process possible								

**Table 3:** TGA analysis to determine the temperatures of 5% and 50% mass loss for four polymers.

Mass Loss	PP	PBS	PBAT	TPS
5%	398.43 °C	304.44 °C	324.73 °C	273.1 °C
50%	440.3 °C	351.53 °C	364.08 °C	354.4 °C

**Table 4:** GPC analysis of three biopolymers before rheology to determine the molecular weight (M_w_), molecular weight distribution (M_n_) and polydispersity.

Polymer	M_w_ [g/mol]	M_n_ [g/mol]	Polydispersity
PBS	6.25 × 10^4^	3.96 × 10^3^	1.59
PBAT	8.14 × 10^4^	5.30 × 10^3^	1.54
TPS	7.26 × 10^4^	2.82 × 10^3^	2.60

**Table 5:** GPC analysis of three biopolymers after rheology to determine the molecular weight (M_w_), molecular weight distribution (M_n_) and polydispersity.

Polymer	M_w_ [g/mol]	M_n_ [g/mol]	Polydispersity
PBS	1.327 × 10^5^	5.304 × 10^4^	2.502
PBAT	9.376 × 10^4^	3.281 × 10^4^	2.858
TPS	8.648 × 10^4^	3.253 × 10^4^	2.659

**Table 6:** Summary of the properties of the different polymer fibers discussed in this study. The polymer samples are prepared as described in Table 2.

Sample	Titer 48 Fibers [dtex]	Titer Single Fiber [dtex]	Tenacity [cN/tex]	Elongation at Break [%]	Overall Drawing	Crystallinity [%]
PBS 1	317	6.6	4.24	446.50	61.71	64.11
PBS 2	426	8.87	4.70	598.40	65.02	64.52
PBS 3	293	6.1	6.83	331.23	65.53	65.77
PBS 4	224	4.67	9.32	265.85	65.48	68.09
PBS 5	218	4.54	9.57	188.97	65.48	67.64
PBS 6	211	4.39	8.39	184.99	103.00	69.56
PBS 7	173	3.6	10.62	136.81	103.50	68.10
PBAT 1	500	10.42	9.13	191.29	55.41	14.84
PBAT 2	530	11.04	8.02	241.76	55.18	13.95
PBAT 3	420	8.75	11.39	149.79	55.68	13.96
PP 1	571	11.89	11.81	569.41	28.93	42.15
PP 2	429	8.93	13.59	422.32	29.24	41.44
PP 3	346	7.21	21.03	276.84	29.75	42.53
PP 4	276	5.75	25.38	185.28	30.26	42.84
PP 5	230	4.79	32.30	137.85	30.75	46.24
PP 6	126	2.62	39.47	104.19	31.52	45.00
PP 7	184	3.83	51.62	59.15	44.04	46.47
PP 8	303	6.31	19.68	402.43	80.97	44.33
PP 9	229	4.77	28.47	250.15	81.39	42.84
PP 10	173	3.6	39.53	141.62	82.00	45.50
PP 11	138	2.87	52.26	62.07	82.66	47.13
TPS		No stable process possible				

**Table 7:** Summary of the parameters determined in this study for four polymers.

Parameter	PP	PBS	PBAT	TPS
Density [g/cm^3^]	0.91	1.26	1.25	>1
Glass transition temperature [°C]	−15	−38	−33	
Melting temperature [°C]	170	113	123	125
Decomposition temperature [°C]	399			
5% Decomposition temperature [°C]	398	304	224	273
Tenacity [cN/tex]	50	10.6	11.3	
Chemical resistance	++	?	?	?
Abrasion resistance	+	?	?	?
UV stability	-	?	?	?
Flame retardancy	-	?	?	?
Titer	2.9	3.6	8.8	-
Elongation at break	59	136	149	-

## Data Availability

The datasets used and/or analyzed during this study are available from the corresponding author on reasonable request.
